# A broader perspective on the economics of malaria prevention and the potential impact of SARS-CoV-2

**DOI:** 10.1038/s41467-022-30273-z

**Published:** 2022-05-13

**Authors:** Elisa Sicuri, Francesco Ramponi, Iris Lopes-Rafegas, Francisco Saúte

**Affiliations:** 1 LSE Health, London School of Economics and Political Science, London, UK; 2 ISGlobal, Hospital Clínic - Universitat de Barcelona, Barcelona, Spain; 3 Manhiça Health Research Center, Manhiça District, Manhiça, Mozambique

**Keywords:** Health care economics, Parasitology, Economics

## Abstract

Economic evaluations of public health interventions to prevent malaria should consider the adoption of wider perspectives and the inclusion of non-health impacts, particularly economic development outcomes, such as education. This is especially relevant in malaria elimination settings and in the context of the current SARS-CoV-2 pandemic.

## The economics of public health interventions to prevent malaria in control and elimination settings

Malaria prevention relies on vector control strategies such as indoor residual spraying and the use of insecticide-treated nets, on preventive chemotherapies such as intermittent preventive treatment in infants and pregnant women, and seasonal malaria chemoprevention in children. In addition, the long-time craved first *P. falciparum* vaccine RTS,S/AS01 has been recently recommended for broad use in children in regions with moderate to high transmission, and initiatives are ongoing to support its rollout in Sub-Saharan African settings. The use of the preventive public health toolbox can aim at either reducing malaria incidence to a locally acceptable level (control) or interrupting local transmission in a defined geographic area (elimination).

In disease control settings, the introduction of safe and efficacious malaria preventive interventions on top of malaria case management activities has typically represented a cost-effective use of health care resources. The same applies when new preventive activities are introduced to complement or replace existing ones, or when these are intensified for improving efficacy: at adequate levels of coverage and adherence, the incremental health impact outweighs the incremental cost^1^.

However, even if preventive tools and strategies are proven to be efficacious in disease modelling exercises or clinical trial settings, their implementation does not always lead to a full realisation of the expected health impacts in real-world settings. Such a gap between efficacy and effectiveness is attributable to both demand- and supply-related factors. Demand-side factors include, for example, lack of awareness of the community benefits of individual preventive choices^2^, inability to pay for the full or even partial cost of the intervention, or difficulties in accessing the preventive activities. Furthermore, when the correct information is lacking, fallacious beliefs based on misleading signals may lead to suboptimal health care choices. For example, in absence of information on treatment, the disappearance of symptoms may cause the interruption of the therapy before its completion, leading to a likely reduction in the preventive effects (i.e. transmission containment) of antimalarial drugs.

Supply-side barriers encompass issues at different levels of the healthcare system such as, for example, products stockouts; poor labour organisation and performance;^3^ and, more generally, funds shortage^4^. Of note, by unfairly affecting access to health care, these barriers to the realisation of the full expected impacts on health may potentially generate health inequities, too.

Innovative mechanisms of delivery and acquisition of preventive tools and strategies of information dissemination have been designed to address at least some of these issues. For example, to improve the coverage of malaria prevention programmes, community health workers and volunteers have been involved in the promotion and distribution of preventive chemotherapies. To improve the coverage and actual use of bed nets, various forms of distribution have been adopted, ranging from free provision, to provision based on cost-sharing^5^ or micro-loans^6^ in search of a balance between ensuring universal ownership and incentivising actual usage. To increase adherence to antimalarial treatment, simple packaging messages have been employed to explain the importance of completing the whole therapeutic course^7^. Most of these strategies are more expensive for the health provider, but have been proven to positively impact health and to be cost-effective. Notably, most of these strategies are also able to improve health equity, by granting access to those that would be otherwise left behind.

In elimination settings, maximising the effectiveness of public health actions to prevent malaria becomes even more critical. This is because the interruption of local transmission requires extra efforts in terms of intensifying and expanding the range of interventions currently implemented. Such “last mile” activities are characterised by high costs, uncertainty and risk of failure. Sources of uncertainty and risk of failure are multiple, such as the lack of political coordination and the difficulties in providing continuous sustained support to policies with a potentially decreasing community demand for prevention in settings where malaria incidence approximates zero. Furthermore, on the path towards elimination, the incremental health impacts decrease. Therefore, if only focused on direct impacts on health and health care costs, cost-effectiveness analysis may discourage investing in elimination policies. For example, non-routine public health interventions, such as mass drug administration (MDA) and reactive focal MDA, together with strengthened surveillance may be essential to achieve and sustain elimination, but economic analyses may indicate that the incremental costs are not compensated by the incremental health benefits in the short-term.

For economic evaluation to be better aligned with decision-making needs and priorities in elimination settings, particular attention should be devoted to two aspects: the consideration of the long-term effects of the policies and their impacts beyond health. To fully reflect the benefits of malaria elimination, it would be necessary to estimate the long-term benefits of a scenario with no malaria. These encompass not only the consequent health benefits and cost savings for the health system, but also the spillovers beyond health and health care. Notably, thanks to the use of robust quasi-experimental methods applied to observational data, malaria public health interventions have been proven to cause, in most of the settings explored, a non-negligible long-term impact on education, labour, income, fertility and, more in general, on economic growth and development^8–10^. However, such evidence is seldom considered in economic evaluations.

To the best of our knowledge, only selected non-health impacts have been included in economic evaluations, specifically in cost-benefit analyses, such as the value of enhanced work productivity or extended productive lifespan and increased revenues from tourism^11^. Moreover, the magnitude of such impacts has been mainly based on assumptions rather than on estimates from robust impact evaluations. For example, the impact on schooling and education outcomes, or more generally on human capital accumulation, has never been explicitly considered in the economic evaluation of public health malaria interventions conducted within the healthcare sector. Such an approach may potentially misrepresent the full impact of malaria prevention interventions^12^. Of note, even if an intervention is deemed cost-effective or cost-beneficial from a narrow health care perspective, the inclusion of potential impacts beyond health in the economic evaluation would provide useful information to decision-makers across different sectors.

While substantial efforts have been made to improve the methods for economic evaluation in the context of disease elimination^13^, the lack of consideration of outcomes falling on sectors other than health may still lead to a subotpimal allocation of resources and to underfunding of key health care interventions for economic development. The adoption of broader perspectives in economic evaluations or the consideration of economic development indicators could lead to policy recommendations that better reflect the wide impact of malaria preventive activities. Even though the measurement of the impact of elimination/eradication of neglected and low transmission diseases beyond health could be challenging, benefits have already been identified relative to education, food security, and happiness^14–16^.

## The impact of the SARS-CoV-2 pandemic on malaria elimination efforts and development

The SARS-CoV-2 pandemic has disrupted malaria healthcare services. According to the latest World Malaria Report, about two-thirds of the 69,000 additional malaria deaths in 2020 compared to 2019 were linked to disruptions in the provision of malaria prevention, diagnosis and treatment during the pandemic^17^.

In settings where elimination activities are being carried out either as part of the national health programmes or as research projects, elimination activities were withdrawn for most of the year 2020. This is the case, for example, in the South of Mozambique where elimination projects, in place since 2015, were interrupted because of mobility restrictions and diversion of healthcare resources from malaria programs to SARS-CoV-2 related activities. For example, community health workers who were meant to be employed in reactive focal MDA were reallocated to address other healthcare priorities. Even if no evidence is yet available for most of the elimination settings, including Southern Mozambique, the interruption of malaria elimination activities is likely to have compromised the fragile health gains in terms of the malaria incidence reduction obtained in the pre-pandemic period^18^. Nevertheless, once available, data will have to be interpreted with caution as reported cases of malaria are likely to have dropped over the year 2020 due to a generalised reduction in the use of healthcare services by the population, as a consequence of governmental recommendation to seek treatment only for the most essential health needs and because of the fear of contracting SARS-CoV-2 infection at the health care facilities.

In addition to the plausible increase in malaria burden, the aftermath of the SARS-CoV-2 pandemic is likely to affect not only health, but also economic development factors. Of particular concern is the impact on education. As in most countries worldwide, schools remained shut for most of 2020 also in Sub-Saharan Africa, with extreme cases such as Uganda, where classes were suspended for almost two years. In malaria endemic settings, school outcomes are likely to have been hit by a double negative shock: school closure and malaria resurgence. The pandemic-induced repercussion of such double negative shock on school outcomes will soon emerge, probably with substantial consequences. School closures are expected to impact education both in the short-term, due to higher absenteeism and lower performance once schools are reopened, and in the long-term, due to definitive school drop-out.

Nevertheless, in endemic areas where elimination activities were successfully carried out before the onset of the pandemic, the decline in short-term school outcomes may be lower than in areas with similar epidemiological and socio-economic conditions, but no malaria elimination activities. In other words, elimination efforts in the pre-pandemic period may have exerted a protective effect against malaria resurgence, and consequently mitigated the worsening of short-term school outcomes. However, in the context of interrupted elimination activities paired with school closures due to SARS-CoV-2, there are potential threats to such mitigation claims. On the one hand, children born in the malaria elimination pre-pandemic years may be at risk of low immunity formation and thus be particularly hit by the potential resurgence of malaria if control/elimination activities are not quickly resumed^19^. On the other hand, as a consequence of the protective effect of elimination campaigns over malaria, improved health in elimination areas may have driven a higher share of children towards the labour market during school closures^20^.

In the near future, it will be fundamental to identify and measure the impact of malaria elimination activities disruption due to the pandemic on education outcomes. This evidence could inform economic evaluations conducted from broader perspectives that could assist decisions on whether, how, and when to resume malaria elimination activities after the SARS-CoV-2 emergency. The generation of such information calls for collaborations across different research fields, including public health, economics, development, and education.

### Reporting summary

Further information on research design is available in the Nature Research Reporting Summary linked to this article.

## Supplementary information

Reporting summary
